# Supplementary material for: Severity of Respiratory Viral Diseases and the Impacts of Underlying Medical Conditions During the Omicron Subvariant Dominant Epidemics—A Comparative Study of SARS-CoV-2, Influenza Virus and Respiratory Syncytial Virus
Source: Pathogens. 2025 May 29;14(6):543. doi: 10.3390/pathogens14060543 (PMC12196169; doi:10.3390/pathogens14060543)
Supplement: Supplementary file 1 [file pathogens-14-00543-s001.zip › pathogens-3597393-supplementary.pdf]

Table S1. Risk of pneumonia development by each underlying disease in patients with severe acute respiratory syndrome coronavirus 2 (SARS-CoV-2)

**Underlying disease**	**SARS-CoV-2**		
	**No (%)**	**OR (95% CI)**	**P-value**
Age		1.03 (1.02–1.04)	<0.001*
Female	183/784 (23.3)	0.76 (0.61–0.96)	0.020*
COVID-19 vaccination	338/1536 (22.0)	0.48 (0.36–0.63)	<0.001*
Diabetes mellitus	210/760 (27.6)	0.48 (0.36–0.62)	0.104
Cardiovascular diseases	105/396 (26.5)	1.07 (0.83–1.38)	0.305
Cerebrovascular diseases	98/240 (29.0)	0.87 (0.66–1.14)	0.742
Neuromuscular diseases	49/123 (39.8)	1.78 (1.20–2.64)	0.004*
COPD	23/78 (29.5)	0.94 (0.55–1.60)	0.816
Asthma	29/63 (46.0)	2.37 (1.39–4.05)	0.002*
Other chronic lung diseases	25/66 (37.9)	1.79 (1.05–3.05)	0.033*
History of tuberculosis	17/75 (22.7)	0.75 (0.42–1.34)	0.329
Chronic renal diseases	68/261 (26.1)	0.99 (0.71–1.37)	0.936
Chronic liver diseases	84/395 (21.3)	0.87 (0.50–1.53)	0.632
Solid cancer	84/395 (21.3)	0.84 (0.63–1.13)	0.260
Hematology malignancy	22/78 (28.2)	0.98 (0.54–1.77)	0.933
Bone marrow transplant	4/6 (66.7)	8.05 (1.27–51.00)	0.027*
Solid organ transplant	5/12 (41.7)	2.55 (0.74–8.88)	0.140
Autoimmune diseases	10/36 (27.8)	1.32 (0.60–2.90)	0.489
Immunosuppressants user	33/125 (26.4)	1.34 (0.82–2.19)	0.238
HIV	2/5 (40.0)	2.81 (0.42–19.00)	0.290

Age was analyzed as a continuous variables.

OR, odds ratio; CI, confidence interval; COPD, chronic obstructive pulmonary disease; TB, HIV, human immunodeficiency virus

* P < 0.05

Table S2. Risk assessment of ICU admission for each underlying disease in patients with severe acute respiratory syndrome coronavirus 2 (SARS-CoV-2)

**Underlying disease**	**SARS-CoV-2**		
	**No (%)**	**OR (95% CI)**	**P-value**
Age		1.00 (0.99–1.01)	0.789
Female	153/784 (19.5)	0.78 (0.61–0.99)	0.038
COVID vaccination	304/1536 (19.8)	0.55 (0.42–0.73)	<0.001*
Diabetes mellitus	185/760 (24.3)	1.16 (0.91–1.47)	0.236
Cardiovascular disease	99/396 (25.0)	1.03 (0.78–1.37)	0.817
Cerebrovascular disease	114/338 (33.7)	1.88 (1.43–2.46)	<0.001*
Neuromuscular disease	36/123 (29.3)	1.38 (0.91–2.11)	0.132
Chronic lung disease	21/66 (31.8)	1.61 (0.92–2.80)	0.095
COPD	20/78 (25.6)	1.14 (0.66–1.98)	0.633
Asthma	12/63 (19.0)	0.71 (0.37–1.39)	0.316
History of TB	18/75 (24.0)	1.07 (0.61–1.90)	0.810
Chronic renal disease	74/261 (28.4)	1.27 (0.92–1.75)	0.154
Chronic liver disease	22/87 (25.3)	1.34 (0.80–2.24)	0.264
Solid cancer	58/395 (14.7)	0.61 (0.44–0.85)	0.003*
Hematology malignancy	11/78 (14.1)	0.57 (0.27–1.20)	0.136
Bone marrow transplant	2/6 (33.3)	2.42 (0.37–15.68)	0.356
Solid organ transplant	5/12 (41.7)	2.77 (0.79–9.72)	0.111
Autoimmune disease	5/36 (13.9)	0.64 (0.24–1.71)	0.372
Immunosuppressants user	17/125 (13.6)	0.75 (0.40–1.37)	0.345
HIV	1/5 (20.0)	0.99 (0.11–9.27)	0.995

Age was analyzed as a continuous variables.

OR, Odds Ratio; CI, Confidence Interval; DM, Diabetes Mellitus; COPD, Chronic Obstructive Pulmonary Disease; TB, Tuberculosis; HIV, Human Immunodeficiency Virus

* $P < 0.05$

Table S3. Risk assessment of mortality for each underlying disease in patients with severe acute respiratory syndrome coronavirus 2 (SARS-CoV-2)

**Underlying disease**	**SARS-CoV-2**		
	**No (%)**	**OR (95% CI)**	**P-value**
Age		1.04 (1.03–1.06)	<0.001*
Female	70/784 (8.9)	0.71 (0.51–0.99)	0.044*
COVID vaccination	133/1536 (8.7)	0.37 (0.26–0.53)	<0.001*
Diabetes mellitus	75/760 (9.9)	0.91 (0.65–1.27)	0.572
Cardiovascular disease	43/396 (10.9)	0.85 (0.58–1.26)	0.424
Cerebrovascular disease	34/338 (10.1)	0.77 (0.51–1.67)	0.220
Neuromuscular disease	13/123 (10.6)	0.89 (0.48–1.66)	0.712
Chronic lung disease	9/66 (13.6)	1.07 (0.50–2.30)	0.853
COPD	8/78 (10.3)	0.67 (0.31–1.48)	0.325
Asthma	9/63 (14.3)	1.25 (0.59–2.67)	0.563
History of TB	10/75 (13.3)	1.16 (0.57–2.40)	0.681
Chronic renal disease	32/261 (12.3)	1.31 (0.84–2.05)	0.228
Chronic liver disease	18/87 (20.7)	2.81 (1.58–5.02)	<0.001*
Solid cancer	58/395 (14.7)	1.83 (1.26–2.66)	0.002*
Hematology malignancy	5/78 (6.4)	0.80 (0.30–2.13)	0.658
Bone marrow transplant	0/6 (0.0)	N/A	N/A
Solid organ transplant	2/12 (16.7)	2.84 (0.55–14.62)	0.212
Autoimmune disease	3/36 (8.3)	1.24 (0.36–4.30)	0.734
Immunosuppressants user	15/125 (12.0)	1.15 (0.60–2.21)	0.683
HIV	1/5 (20.0)	4.64 (0.45–47.62)	0.197

Age was analyzed as a continuous variables.

OR, Odds Ratio; CI, Confidence Interval; DM, Diabetes Mellitus; COPD, Chronic Obstructive Pulmonary Disease; TB, Tuberculosis; HIV, Human Immunodeficiency Virus; N/A, Not Available

* P < 0.05
